# Metabolic Targets for Treatment of Autoimmune Diseases

**DOI:** 10.20900/immunometab20200012

**Published:** 2020-03-31

**Authors:** Paramarjan Piranavan, Manjeet Bhamra, Andras Perl

**Affiliations:** 1 Department of Medicine, College of Medicine, State University of New York, Syracuse, NY 13210, USA; 2 Department of Microbiology and Immunology, College of Medicine, State University of New York, Syracuse, NY 13210, USA; 3 Department of Biochemistry and Molecular Biology, College of Medicine, State University of New York, Syracuse, NY 13210, USA

**Keywords:** mechanistic target of rapamycin, immune metabolic pathways, systemic lupus erythematosus, rheumatoid arthritis, psoriasis, scleroderma, pentose phosphate pathway, tryptophan, kynurenine, glycolysis, oxidative phosphorylation, oxidative stress, acetylcysteine

## Abstract

There is a considerable unmet demand for safe and efficacious medications in the realm of autoimmune and inflammatory diseases. The fate of the immune cells is precisely governed by control of various metabolic processes such as mitochondrial oxidative phosphorylation, glycolysis, fatty acid synthesis, beta-oxidation, amino acid metabolism, and several others including the pentose phosphate pathway, which is a unique source of metabolites for cell proliferation and maintenance of a reducing environment. These pathways are tightly regulated by the cytokines, growth factors, availability of the nutrients and host-microbe interaction. Exploring the immunometabolic pathways that govern the fate of cells of the innate and adaptive immune system, during various stages of activation, proliferation, differentiation and effector response, is crucial for new development of new treatment targets. Identifying the pathway connections and key enzymes will help us to target the dysregulated inflammation in autoimmune diseases. The mechanistic target of rapamycin (mTOR) pathway is increasingly recognized as one of the key drivers of proinflammatory responses in autoimmune diseases. In this review, we provide an update on the current understanding of the metabolic signatures noted within different immune cells of many different autoimmune diseases with a focus on selecting pathways and specific metabolites as targets for treatment.

## INTRODUCTION

The role of the metabolic pathways in growth, proliferation and survival of prokaryotic and eukaryotic organisms has long been recognized. Many decades ago, Warburg elucidated the importance of metabolism in controlling cancer development and persistence by highlighting the shift to glycolysis from mitochondrial oxidative phosphorylation [1]. Metabolic pathways govern the lineage specification of immune system by regulating the glucose utilization to generate adenosine triphosphate (ATP) molecules and synthesize amino acids, nucleotides and lipids to meet the demands of various immune cells. Evading microbes, malignancies, and other environmental foreign antigens constantly challenges innate and adaptive system. Prompt response of immune cells with massive proliferation, migration to specific tissue sites and synthesis of effector molecules, achieved by rapid generation of energy from metabolic shifts. Understanding the metabolic control over normal immune response will help us to explore the dysfunctional metabolic shifts in autoimmunity. Various disease specific derangements in metabolic pathways are, identified in lymphocytes derived from systemic lupus erythematosus (SLE) and rheumatoid arthritis (RA) patients [2]. With consideration to immune cells, the metabolic signature is known to change depending on stages of development and pathological conditions, whether they are in quiescent, activated, or, memory state [3–5]. In this review, we provide an update on the current understanding of the metabolic signatures noted within different immune cells of many different autoimmune diseases with a focus on selecting pathways and specific metabolites as targets for treatment.

## PHARMACOLOGIC TARGETING OF METABOLIC PATHWAYS IN THE RHEUMATIC DISEASES

With the development of more unifying treatment guidelines with early, goal directed treatment, and usage of synthetic/biologic Disease modifying anti rheumatic drugs (DMARDs); RA has become a treatable disease with favorable outcome [6]. However there are other rheumatic diseases like SLE, Scleroderma where we have few FDA approved drugs available as treatment options [7,8]. Majority of the synthetic DMARDs available in market to date or under development, targets various metabolites in immune cells as listed below in Table 1. Although many biologic DMARDs and biosimilars have become available in last 2 decades, they remain expensive, and have increased the cost of health care [9]. Biologics may have relatively quick onset of action than synthetic DMARDs, but there is no convincing superior clinical efficacy demonstrated over them [10].

**Figure 1.**
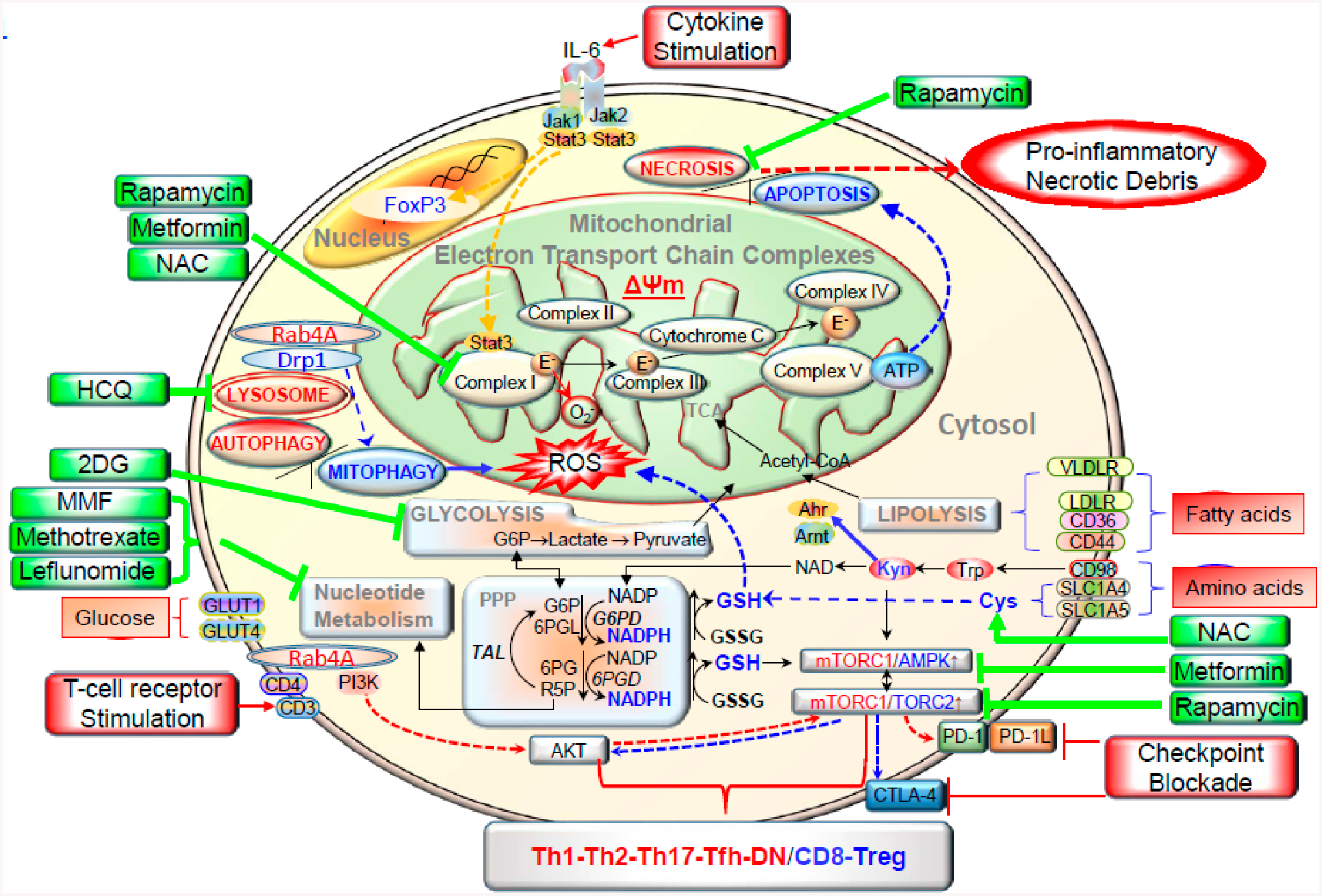
This schematic diagram shows the metabolic pathways controlling activation and lineage specification in the immune system. Depicts how various metabolic pathways regulate the surface receptors, and intracellular transducers in immune cells. Electron transport chain blockage results in elevated mitochondrial transmembrane potential (Δᴪm) or mitochondrial hyperpolarization, and diminished mitophagy contributes to accumulation of oxidative stress-generating mitochondria and depletion of ATP and glutathione. Reactive oxygen species (ROS) are generated by electron transfer to O_2_ at complex I. These metabolic changes underlie the activation of mTORC1, which promotes glycolysis in CD4^+^ T cells, further enhancing accumulation of mitochondria in necrosis prone, pro inflammatory double negative (DN) T cells and depleting Treg cells. The direction of signaling is indicated by arrow (red = increase, blue = decrease). Drugs that affect metabolism are shown in green. IL 6 = interleukin-6, NAC = *N*-acetyl cysteine; Drp1 = dynamine-related protein 1; HCQ = hydroxychloroquine; TCA = tricarboxylic acid; 2 DG = 2 Deoxy glucose; Acetyl-CoA = acetyl co enzyme A; VLDLR = very low density lipoprotein receptor; MMF = mycophenolate mofetil; LDLR = low density lipoprotein receptor; G6P = glucose-6-phosphate; PPP = pentose phosphate pathway; G6PD = glucose-6-phosphate dehydrogenase; 6PGL = 6-phosphogluconolactonase; GSSG= oxidized glutathione; TAL = transaldolase; 6PG = 6-phosphonogluconate; 6PGD = 6-phosphogluconate dehydrogenase; AMPK = AMP dependent protein kinase; PI3K = phosphatidylinositol 3 kinase; R5P = ribose-5-phosphate; PD-1 = programmed death 1;Tfh = follicular helper T cells. Reproduced from [12], an open access article distributed under the Creative Commons Attribution 4.0 International License (http://creativecommons.org/licenses/by/4.0/).

That tells us that we need to keep exploring the metabolic targets in immune cells to change the favorable outcome of many rheumatic diseases. Figure 1 broadly outlines the metabolic pathways controlling inflammatory lineage specification of immune system in SLE and other autoimmune diseases. To further delineate we are going to explore the normal and abnormal metabolic pathways in immune cells together highlighting the potential targets in those individual pathways. We also will summarize the various receptors and intracellular transducers like mammalian target of rapamycin (mTOR) [11], adenosine monophosphate activated protein kinase (AMPK), aryl hydrocarbon receptor (AhR), general control nonderepressible 2 (GCN2) that are also controlled and closely linked to metabolic pathways, as drugs targeting them shown some promising outcome in various trials.

## GLUCOSE METABOLISM

Both resting and activated lymphocytes primarily rely on glucose for their ATP synthesis [40]. Following the glucose entry in to cell via glucose transporters, hexokinase phosphorylates it to glucose-6-phosphate to prevent its leakage. Glucose-6-phosphate then can commit to either glycolysis or pentose phosphate pathway (PPP). Glycolysis is anaerobic and generates 2 molecules of ATP, pyruvate, and reduced nicotinamide adenine dinucleotide (NADH). Fate of pyruvate depends on the oxygen availability and type of cell, (resting, memory or activated immune cells). In resting cells with oxygen abundance, pyruvate enters tricarboxylic acid (TCA) cycle and generates NADH, reduced flavin adenine dinucleotide (FADH) molecules. They enter electron transport chain and through oxidative phosphorylation (OXPHOS) synthesize around 30–36 ATP molecules [41]. PPP generates ribose-5 phosphate necessary for nucleotide synthesis and produce the essential NADPH for antioxidant defenses. Immune cells make a choice between speed and efficiency in selecting the suitable pathway for their glucose molecule, based on their demands and needs. For example, when activated by antigens, it has been shown that glycolysis can take over as the primary source of ATP in naïve cluster differentiation (CD) 4 or CD8 T cells [42,43]. However, in chronic activated states (after approximately 5 weeks of chronic self-antigen activation), lupus CD4 T cells have demonstrated an increase in OXPHOS as an ATP source, much like what it seen in thymic, quiescent, memory and regulatory T cells [44–46].

## A—TARGETS IN GLYCOLYSIS

Inflamed joints, inflammatory sites are scarce of oxygen, resulting in production of hypoxia inducible factor-1-alpha (HIF 1α) and in RA promote angiogenesis, inflammation, oxidative damage and cartilage erosion [47]. Studies have demonstrated glycolytic dependent pathway serves as the metabolic checkpoint for Th17, Treg cells [48] differentiation and critical in regulating the transcription of Interleukin (IL)-10 in B cells [49], and glucose transporter (GLUT) 1, lactate dehydrogenase (LDH) up regulation in fibroblast like synoviocytes (FLS) [50]. HIF 1α expression is increasingly observed in lupus nephritis patients than controls, in both glomeruli and tubulointersitial regions. Interestingly they also correlate with SLE disease activity index (SLEDAI) score, renal pathology activity index and proteinuria [51]. HIF 1α inhibitors are in the early stages of clinical trial in the field of RA [47].

Hexokinase 2 is another glycolysis regulator and predominantly expressed in FLS in RA joint and mouse studies have shown inhibiting downstream of HK2 by 2-deoxy glucose (2DG) results in reduction in auto immune model of RA [50]. In CD4^+^ T cells in mice, 2DG along with metformin restores defective IL-2 production and reduce disease activity [15]. 6-Phosphofructo-2-kinase/fructose-2,6-bisphosphatase isoenzyme 3 (PFKFB3) is one of the most important glycolytic enzymes that mediate Warburg effect in tumor and inflammatory cells [52]. There are four isoenzymes (PFKFB 1–4), and compared to the rest, PFKFB3 has the highest kinase/phosphatase ratio. Studies done in naïve CD4 T cells from RA patients have shown that PFKFB3 deficiency known to impair redox balance, autophagy, and ATP generation [52]. Relative PFKFB3 deficiency in RA T cells results in decreased glycolytic activity and increased PPP pathway resulting in NADPH production [52]. Reduced environment and low reactive oxygen species (ROS) in RA T cells result in insufficient activation of the redox sensing kinase ATM [53]. ATM deficiency promotes RA naïve T cells to differentiate in to pathogenic Th1 and Th17 cells and this phenomenon is stopped by replenishment of ROS [54]. On the contrary, PFKFB3 expression is increased in synovial tissue and fibroblast-like synoviocytes in RA patients compared to osteoarthritis patients.[55] Blockade of PFKFB3 pathway resulted in reduced expression of IL-6, IL-8, CCL-2, CXCL-10 and decreased proliferation, migration and invasion of FLS in RA patients [55]. Metformin, an AMPK activator, could be potentially used in RA patients as *N*-myristoyltransferase deficiency in RA T cells, impaired activation of AMPK and promoted synovial inflammation [56].

## TARGETING LACTATE BUILDUP IN THE SETTING OF CHRONIC INFLAMMATION

The role of lactate in modulating the function of tissue resident immune cells in the setting of inflammation and tumor microenvironment has been recently recognized [57,58]. Pucino et al. showed that lactate accumulation in the tissues with chronic inflammation facilitates the up-regulation of lactate transporter SLC5A12 in CD4 T lymphocytes. Uptake of lactate into CD4 T cells via SLC5A12 resulted in Th17 differentiation evident by increased RORγ signature and elevated Th17 cytokines (IL-17 and IL-22) [58]. Monoclonal antibodies against SLC5A12 were successfully used to treat murine model of arthritis, and SLC5A12 is a potential target for future human trials for RA and other inflammatory arthritis [58].

## B—TARGETS IN OXPHOS

Metformin can has elicit immunosuppressive effect by activation of AMPK, an inhibitor of mTORC1 and inhibit mitochondrial complex 1 activity [59]. Pioglitazone a peroxisome proliferator-activated receptor (PPAR)-γ inhibitor, exhibit anti-inflammatory, anti-oxidant, anti-mTORC1 effect by interacting with iron-binding protein to external mitochondrial membrane termed as mitoNEET [28]. Mitoquinone is an antioxidant, which contains ubiquinol and targets by interacting with complex II. In SLE, it reduces reactive oxygen species (ROS) production; oligomerization of mitochondrial antiviral signaling protein (MAVS) in T cells and reduces type 1 interferon production in plasmacytoid dendritic cells (pDC) [27]. 2-(3-Pyridinyl)-1-hydroxyethylidene-1,1-phosphonocarboxylic acid (3-PEHPC) blocks rab geranylgeranyl transferase and reverses the depletion of dynamin related protein (Drp) 1 and accumulation of mitochondria in lymphocytes, macrophages and inhibit ANA production in mice with lupus nephritis (LN)[37].

## C—TARGETS IN PENTOSE PHOSPHATE PATHWAY

Comprehensive metabolome analysis identified the PPP, as most impacted by SLE, along with glutathione, mitochondrial fatty acid and tryptophan metabolism [45]. The enzyme transaldolase (TAL) controls the PPP via controlling the production of reduced glutathione in human T cells. Enhanced mitochondrial metabolism, such as mitochondrial hyperpolarization [3] and block in electron transport [4] occur with increased production of reactive oxygen intermediates (ROI) and the depletion of antioxidant metabolites, such as reduced glutathione [3]. The rate-limiting constituent of de novo reduced glutathione (GSH) production is cysteine, which can be replaced with a cell-permeable precursor, *N-*acetyl cysteine (NAC) [5]. NADPH that is required to maintain GSH in reduced form was also depleted in SLE and more over cysteine itself was depleted in lymphocytes of SLE patients that provide a clear rationale for its therapeutic replacement [60]. A double-blind placebo-controlled pilot study showed that restoration of intracellular GSH with NAC corrected T-cell dysfunction, such as depletion of regulatory T cells and reduced anti-DNA production [5].

Contrary to SLE, in rheumatoid arthritis ROS and oxidative burst offers protection from arthritis and reduced ROS production, NADPH oxidase (NOX) 2 deficiency associated with increased severity of joint inflammation [61]. Depletion of GSH can activate mTOR and studies targeting rapamycin have proven safety and efficacy in SLE [62].

## LIPID METABOLISM

Autoimmune diseases like RA, SLE and psoriatic arthritis (PsA) is associated with hyperlipidemia and studies have shown increased SLEDAI score and anti-ds-DNA antibody titers in SLE patients with elevated circulating triglycerides and total cholesterol [63,64]. Fatty acid acids provide energy through beta-oxidation and also functions as major components of membranes, organelles such as endoplasmic reticulum and golgi body [65]. Studies also have demonstrated that saturated fatty acids promote inflammation and polyunsaturated fatty acids are anti-inflammatory in nature [65]. In RA patients, sphingosine-1-phosphate (S1P) blockage suppresses the matrix metallopeptidase-9 productions in peripheral blood mononuclear cells and down regulation of S1P limited synovial inflammation and pathology [66]. In vitro studies have shown that inhibiting the metabolism of glycosphingolipid biosynthesis can lead to decreased anti-double stranded DNA antibody production in SLE patients [67].

Podosome scaffolding protein TKS5 increases the invasive capabilities of RA T lymphocytes by forming tissue-invasive membrane structures [68]. TKS5 overexpression is driven by reduced glycolytic flux, increased fatty acid synthesis and reduction in fatty acid synthesis reverses it. PFKFB3 blockade increase the TKS5 expression and fatty acid synthase inhibitors reduced the expression [68]. Glycolytic activity and fatty acid synthesis are the key metabolic regulators of controlling the locomotion of the pathogenic T cells and these metabolic checkpoints are potential future targets for RA therapeutics.

## TARGETS IN LIPID METABOLISM

In clinical trials, omega-3 supplementation has also been helpful for ameliorating patient centered outcomes in SLE patients (RAND SF-36) showing another metabolic pathway, which can be utilized for treatment [69]. In Lupus prone mice fingolimod, a S1P receptor inhibitor that interferes with the exit of lymphocytes of lymph node effectively suppressed the development of autoimmunity and extended the lifespan [38]. It is also a FDA approved and one of the effective treatment options for multiple sclerosis [70]. Another novel drug dihydrosphingosine-1-phosphate also has shown that it may serve as anti-fibrotic agent in systemic sclerosis patients [39]. Fatty acid synthase, ATP citrate lyase, acetyl-coenzyme A carboxylases, choline kinases, monoglyceride lipase, 3-hydroxy-3-methyl-glutarylcoenzyme A (HMG CoA) reductase plays important role in tumors and might be a potential targets in rheumatic diseases [71].

## AMINO ACID METABOLISM AND POTENTIAL TARGETS

**Figure 2.**
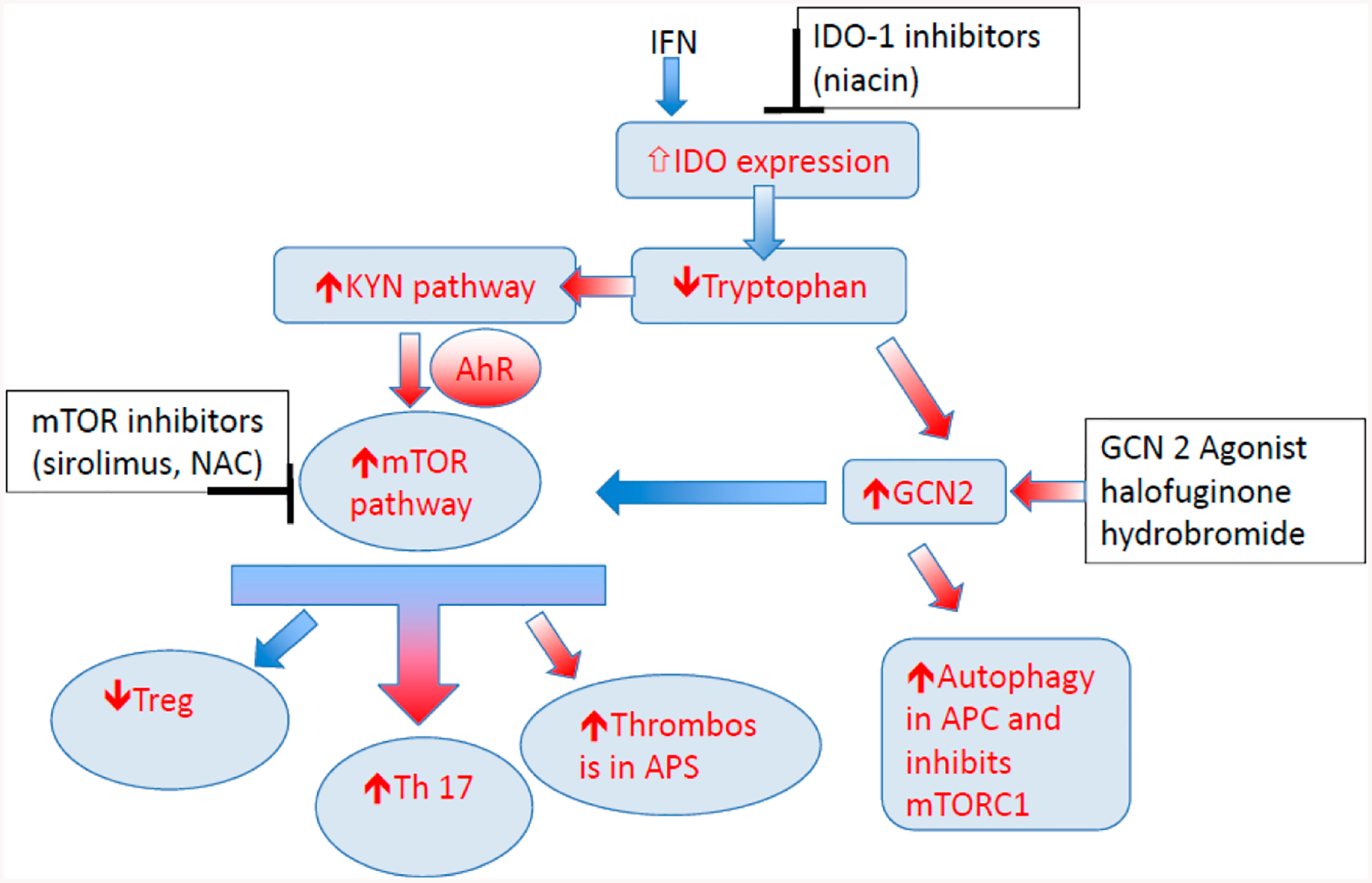
Metabolic control of T-cell related to tryptophan metabolism. Inflammatory cytokines like interferon known to enhance expression of key enzyme IDO. This results in tryptophan depletion as majority of the resident tryptophan is metabolized via KP, and increase in KYN. Depletion of Tryptophan activates the GCN2 kinase pathway and inhibits mTOR pathway. KYN can enhance AhR/mTOR activity, and may increase the risk of thrombosis in anti-phospholipid syndrome. KYN also through AhR/mTOR can increase Th17 differentiation and block Treg differentiation. GCN2 kinase pathway can inhibit mTOR activity and enhance autophagy. IDO inhibitors, mTOR inhibitors, and GCN2 agonist are some of the potential targets in this pathway that can be utilized to treat autoimmune diseases. Note: KYN = Kynurenine, KP = KYN pathway; IDO = indoleamine 2,3-dioxygenase; AhR = aryl hydrocarbon receptor; mTOR = mammalian target of rapamycin; GCN 2 = General Control Nonderepressible; TNF = tumour necrosis factor, IL = interleukin; IFN = interferon.

Amino acids not only serve as nutrient source for T cells but also take part in protein and nucleic acid biosynthesis. It was shown that adjustments to amino acid metabolism within granulocytes could specifically prevent autoimmune signatures. Metabolic stress and nutrient deprivation in immune cells resulting in increased catabolism in lysosomes and glutamine, branched amino acids, kynurenine (KYN) and histidine accumulation. This can result in activation of the mTOR complex [12]. Glutamine uptake is significantly increased upon activation of T cells [72]; at times its uptake is comparable to glucose [73]. In addition in lymphocytes, glutamine contributes to protein, fatty acid and nucleotide synthesis and redox control [61]. Indoleamine-2,3-dioxygenase 1 (IDO1), Arginase 1 (ARG1), the main catabolizing enzymes in tryptophan and arginine metabolism are studied in autoimmune diseases. Potential anti-inflammatory targets in the tryptophan metabolism are highlighted in Figure 2.

Inhibiting tryptophan catabolism via IDO1 was able to protect both human (in vitro) and mice (in vivo) kidney podocytes from antibody-mediated injury [74]. Moreover, giving an IDO1 inhibitor to SLE model mice was able to potentiate SLE phenotype faster [75]. Overexpression of ARG1 is seen in significant proportion of psoriatic patients [76], and ARG1 inhibitors can be potentially used to treat psoriasis in those who overexpress [77].

## REGULATION OF AMINO ACID METABOLISM BY INTERACTING AMPK, GCN2, AND MTOR PATHWAYS

GCN2, mTOR and AMPK are the major amino acid sensors within the cell [78,79], however the way they coordinate these sensing is completely elucidated [80]. Starvation, amino acid depletion typically activates GCN2, AMPK and promotes autophagy, whereas repletion of amino acids and nutrient rich state activates mTOR pathway which plays a major role in protein homeostasis by promoting protein synthesis and inhibiting autophagy [81,82]. In mice hepatocytes, both insulin and amino acids increased mTOR phosphorylation reduced GCN2 phosphorylation, whereas AMPK phosphorylation reduced by high amino acid concentration with or without insulin [78]. Moreover the activation of GCN2 is thought to lead to drive efficient autophagy particularly within granulocytes and other antigen presenting cells (APC) and subsequently lead to an anti-inflammatory state [83]. Inhibition of inflammatory responses to apoptotic cells and development of autoimmunity, were demonstrated through GCN2 [84]. In Lupus prone mice, GCN 2 deletion resulted in severe autoimmunity whereas GCN 2 agonist reduced ds-DNA antibodies and protected mice from sever flare [84].

GCN2, akin to sirolimus, is also known to be involved in the process of inhibiting mTORC1 in the setting of leucine or arginine deprivation states within mouse fibroblasts [85]. Apart from amino acid deprivation states, sirolimus is also known to potentiate GCN2 activity within yeast cells adding to an additional mechanism of controlling SLE disease activity [86]. Moreover, use of a not-so-well known anti-inflammatory agent halofuginone hydro bromide [87] functions as a GCN2 agonist which has been mentioned to drive “potent suppression of lupus” [88].

## KYNURENINE AS A POTENTIAL TARGET IN TRYPTOPHAN METABOLISM

The most robustly accumulated metabolite in SLE peripheral blood lymphocytes is kynurenine [45,89]. Kynurenine is capable of activating mTORC1 in pro-inflammatory, CD4^−^CD8^−^ double-negative T cells [45]. With respect of matched control subjects, comprehensive mass spectroscopy studies identified the accrual of KYN as the most predictive metabolic biomarker of SLE that was also responsive to treatment with NAC [60]. Analyses have been done in human peripheral blood lymphocytes in SLE patients to correlate SLEDAI score with mitochondrial metabolic signatures. KYN itself was also found to activate mTOR, suggesting that metabolites affect lineage specification during T-cell development [62]. Being a natural ligand of the AhR [90], KYN may serve as a signal transducer of Th17 development and inhibitor of Treg differentiation [91]. KYN also inhibits autophagy and promotes senescence [90], which is consistent with mTOR [92] and its role in aging, inflammation and carcinogenesis [11]. Of note, KYN also promotes thrombosis in cancer models [93]. Given the newly uncovered role for mTOR in production of antiphospholipid antibodies outside [94] or within the context of SLE [95,96], KYN should be considered a metabolic trigger of thrombosis through the tissue factor-plasminogen activator inhibitor 1 axis (Figure 2) [93].

## CGAS-STING-IFN PATHWAY TARGETING IN SLE

**Figure 3.**
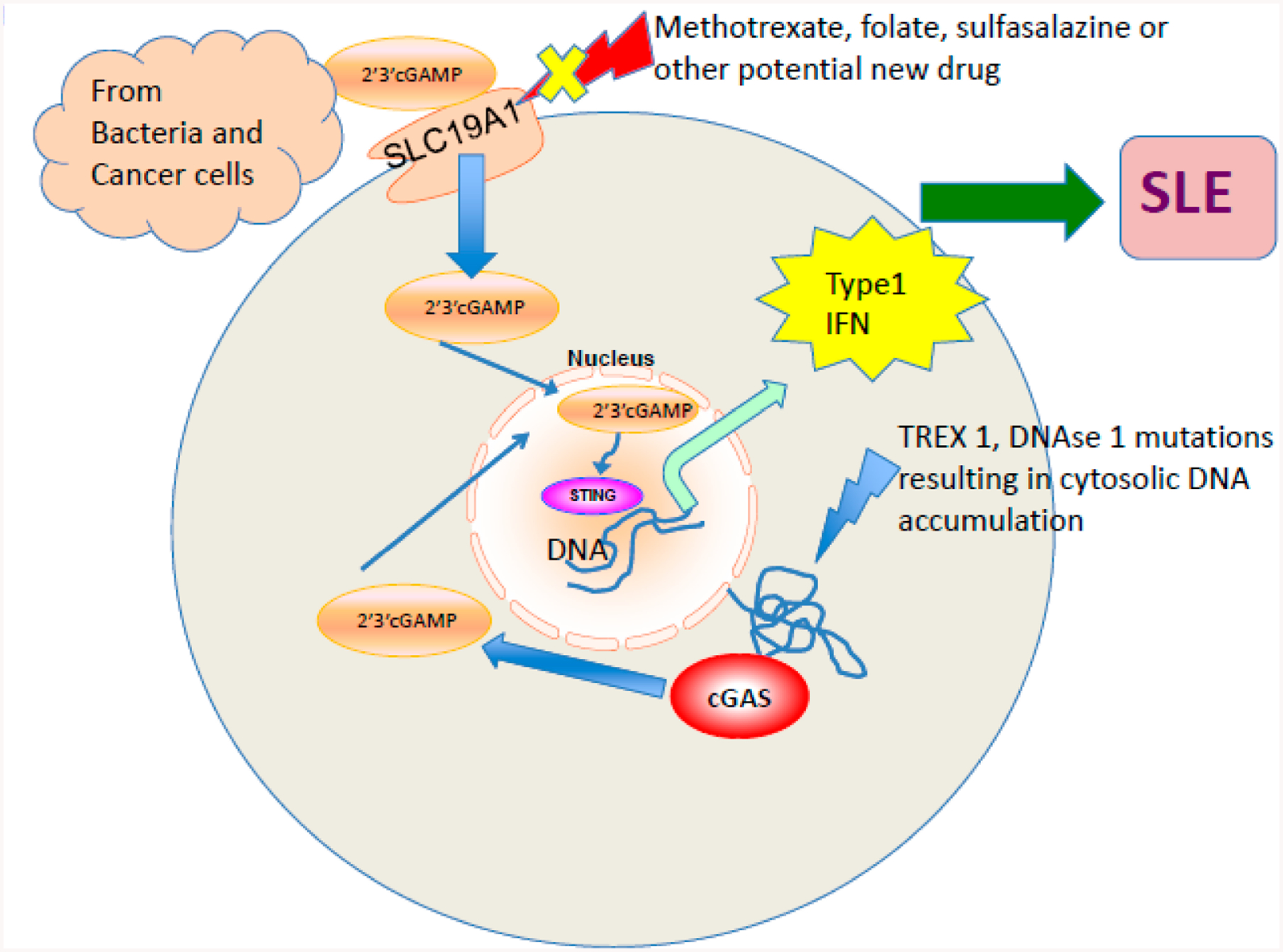
cGAS-STING-IFN pathway activation in SLE. The exogenous cyclic GMP–AMP dinucleotide (2′3′cGAMP) released by bacteria or tumors is imported through the reduce folate career transporter SLC19A1 into the cell. cGAS binds to cytosolic DNA and results in endogenous cGAMP production. Both the exogenous and endogenous cGAMP binds to STING adapter protein and induces transcription of type 1 interferon, the major mediator in SLE pathogenesis. TREX 1, DNAse 1 mutations in humans, results in DNA accumulation and increased susceptibility to SLE like syndromes. SLC19A1 is a potential target to control SLE disease activity and existing drugs like folate, methotrexate, and sulfasalazine known to inhibit it in studies.

Although the importance of type 1 interferon (IFN), interferon signature in SLE was elucidated long ago, the mechanism driving interferon production is not clearly understood [97]. Toll like receptor-9, and cyclic guanosine monophosphate—AMP (2′3′cGAMP) synthase (cGAS)-stimulator of interferon genes (STING) are crucial DNA sensing pathways implicated in the pathogenesis of SLE via IFN production (Figure 3) [97,98]. Recent studies have identified expression of cGAS in peripheral blood mononuclear cells is higher in SLE patients than controls, and around 15% of SLE patients had measurable 2′3′cGAMP levels [99]. As depicted in Figure 3, the binding of DNA to cGAS, leads to endogenous second messenger 2′3′cGAMP production, which activates STING adapter protein that induces IFN transcription [100–103]. Mutations of TREX 1 exonuclease, and DNAse I resulting in defecting endogenous degradation are known to cause SLE and SLE like syndromes with elevated IFN signature (ISG) in humans [102,103]. Exogenous 2′3′cGAMP produced by pathogens, malignant cells enter cell through a recently identified transporter SLC19A1 (a reduce folate career) activate STING and increase IFN production [101]. Studies have shown, available drugs like folate, methotrexate and sulfasalazine inhibit cGAMP uptakes in cells [101]. More potent inhibitors of metabolite transporter SLC19A1 may be potential treatment option in SLE, particularly patients with high ISG.

## THERAPEUTIC MTOR PATHWAY BLOCKADE IN AUTOIMMUNITY

**Figure 4.**
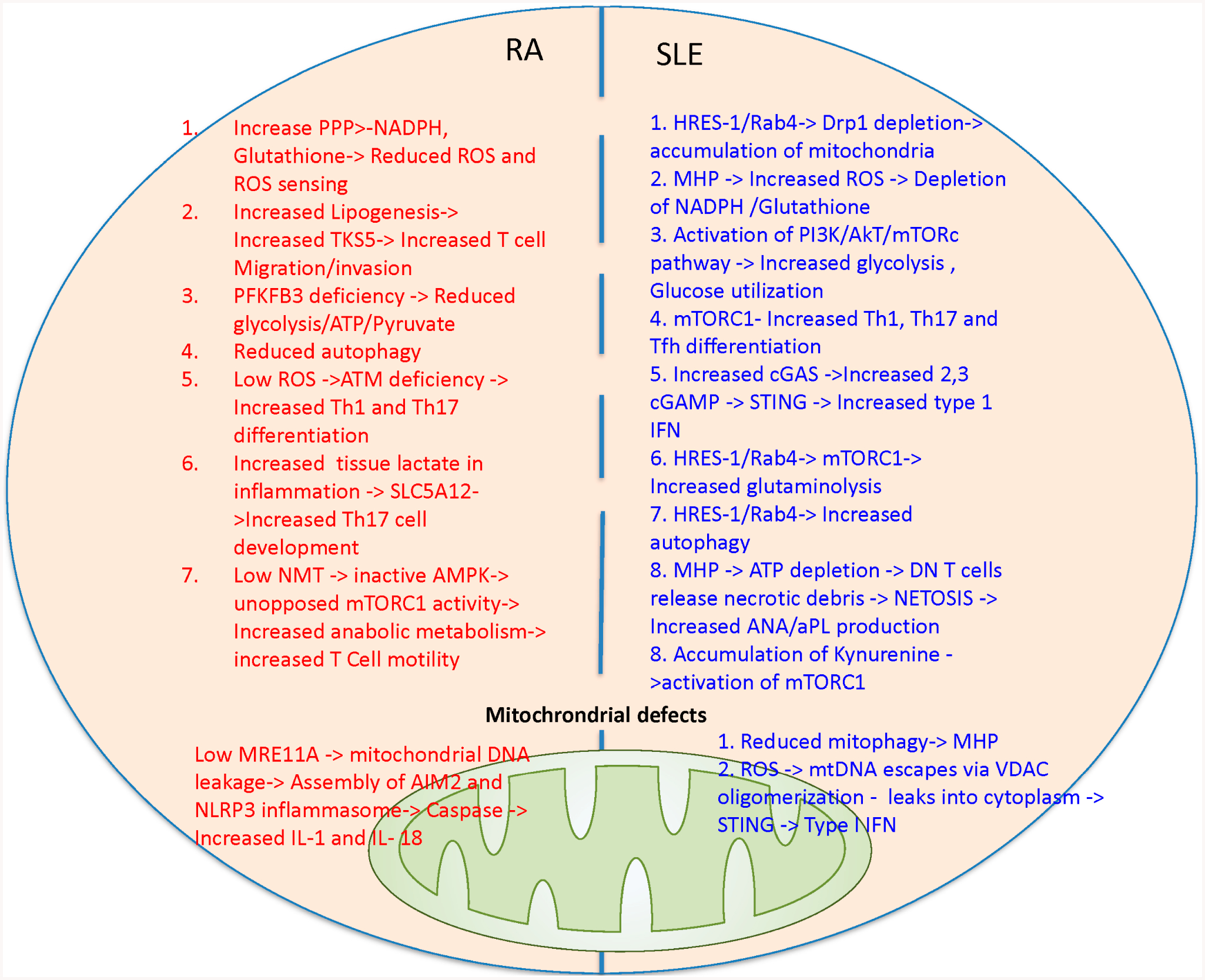
Discordant changes in mitochondrial metabolic pathways in patients with RA and SLE. In RA T cells glucose is shunted more towards PPP pathway leading to increased NADPH and reduced ROS production and sensing that underlie ATM deficiency. In turn, ATM deficiency promotes pathogenic Th1 and Th17 differentiation and production of inflammatory cytokines. Reduced glycolysis in RA T cells is attributed to PFKFB3 deficiency and thus fatty acid synthesis and TKS5 production are increased. TKS5 helps in T cell invasion and migration. Low NMT in RA T cells result in low AMPK activation leading to unopposed mTORC1 activation. Increased glycolysis and lactate uptake via SLC5A12 elicit Th17 expansion. Low MRE11A in mitochondria can lead to mitochondrial DNA leakage resulting in activation of inflammasome (AIM2 and NLRP3) and caspase-dependent production of IL-1 and IL-18. In SLE T cells, genetically enforced increased expression of HRES-1/Rab4 causes depletion of Drp1, reduced mitophagy, accumulation of oxidative stress-generating mitochondria [37] and activation of mTORC1 [121]. Oxidative stress will result in NADPH and glutathione depletion. mTORC1 activation leads to increased glycolysis, glucose utilization and facilitates Th1, Th17 and Tfh differentiation. Increased endogenous cGAMP produced via cGAS and exogenous cGAMP uptaken via SLC19A1 can activate STING and promotes type I interferon production. Accumulation of kynurenine also activates mTORC1. mTOR activation and oxidative stress are most prominent in double negative (DN) T cells that release necrotic debris [29]. Accumulation of mitochondrial occurs with mitochondrial hyperpolarization that results in ATP depletion and increased ROS production [44]. Mitochondrial DNA can leak via voltage dependent anion channels (VDAC) oligomerization can increase type 1 interferon production.

The mTOR has been shown to be a target of therapy in both lupus model mice [37] and SLE patients [29,30,96]. Recently, there has been further work to distinguish the specific role subtypes of mTORC1 and mTORC2. Both mTOR complexes are understood to shift T follicular helper cells metabolism towards glycolysis/anabolic metabolism. Understanding that T follicular helper cells are culprit in SLE, targeting metabolic pathways in T follicular helper cells is a potential target [104]. mTORC1 is known to act upstream of a protein Raptor and mTORC2 is known to act upstream of a protein Rictor. When studying the Peyer’s patches (known to contain more memory T cells as compared with the T cells in the spleen) of knockout mice models of these specific genes (Raptor and Rictor), it was shown that inhibiting mTORC2 pathway specifically results in decreased IgA+ B Cells via flow cytometry, which is not observed by mTORC1 inhibition. It was also shown in the same work that inhibiting both mTOR complexes resulted in a reduced frequency of T-follicular helper cells in flow cytometry of Peyer’s patches from wild type, Raptor negative, and Rictor negative knockout mice. mTOR blockade with sirolimus has been shown to stop the development of autoantibodies from BAFF stimulated B cells in in-vitro mice splenic B-cell cultures [105]. In SLE model mice, sirolimus was shown to help reduce the development of anti-dsDNA antibodies as well as proteinuria [32]. Furthermore, a phase 2 trial of sirolimus in human SLE patient’s showed a statistically significant decreased in British isles lupus activity group (BILAG) scores after 1 year of treatment in an open label single-arm study [96]. Short term sirolimus (3 days), which is understood to exclusively cause mTORC1 blockade helped increase transforming growth factor (TGF)-Beta that is known to have anti-inflammatory effects in SLE. Longer term treatment with sirolimus (4 weeks), which is understood to cause blockage of both mTORC1 and mTORC2, was able to restore normal gene expression of GATA-3 and cytotoxic T lymphocyte associated antigen (CTLA-4) in Treg cells as measured by flow cytometry in human peripheral blood mononuclear cells [106]. This ultimately suggests more longstanding benefits of long-term sirolimus administration. The treatment with NAC blocked the activation of mTORC1 that appears to be exceedingly high in double-negative T cells [5], which produce pro-inflammatory cytokines, such as IL-4 and IL-17 [107]. In contrast to SLE, mitochondrial oxidative stress appears to be reduced in RA due to increased NADPH production through the PPP, as shown in Figure 4.

Another disease model to study with regards to mTOR complex functioning is tuberous sclerosis (TSC). According to the NIH, tuberous sclerosis is an autosomal dominant genetic disorder that ultimately leads to benign tumor growth in multiple organs of the body and it is cardinally seen with mutations in either TSC1 or TSC2. TSC1 and TSC2 are a part of a complex in mice that, when not present such as in tuberous sclerosis, lead to uncontrolled mTORC1 activation [108]. Specifically, in the radial glial cells of knock out mouse models of tuberous sclerosis (TSC2-RG mice) metabolic shifts towards anabolic pathways with increased glutaminolysis and pentose phosphate pathway activity were both noted in tuberous sclerosis mice models. Moreover, weight discrepancies that developed between wild type and tuberous sclerosis mice models resolved with sirolimus administration [109]. This model for studying mTOR has clinical implications as well given that patients with tuberous sclerosis mutations have been described to develop SLE like disease [110,111].

## MONOCYTE METABOLIC PROFILE/SIGNATURES IN AUTOIMMUNE DISEASES

When compared to resting cells, human and murine macrophages are generally known to increase glycolytic rate and reduce OXPHOS upon activation [112]. However, metabolic changes differ according to the macrophage phenotype. M1 macrophages use glycolysis as their primary energy source and M2 macrophage use OXPHOS as primary source for long and sustained response [112,113]. Macrophages derived from RA and coronary artery disease (CAD) patients have shown evidence of hypermetabolism, increased glucose utilization and increased ATP production by upregulation of glucose transporters and major glycolytic enzymes. Increased OXPHOS and oxidative stress in macrophages will result in oxidized pyruvate kinase M2 (PKM2). Oxidized PKM2 when dimeric can enter the nucleus and moonlights as a protein kinase by phosphorylating STAT3. This results in increased production of IL-1β and IL-6, which are major drivers of atherosclerosis and synovial inflammation [57]. Watanabe et al. compared the metabolic profile of macrophages derived from vessel wall in CAD and giant cell arteritis (GCA) patients. Macrophages from GCA patients were like healthy controls and had normal glucose utilization, and cytokine production. However, macrophages from CAD showed up regulation of glycolytic enzymes, with increased glucose utilization and cytokine production as compared to healthy controls and GCA patients [114].

Defective phagocytic ability, surface marker expression and cytokine production by both macrophages and monocytes were demonstrated in SLE [113,115]. Type 1 interferon signature is increased in monocytes and macrophages in SLE [113]. Korman et al. identified differential expression of lipid and carbohydrate metabolism genes in peripheral blood monocytes derived from 20 SLE patients compared with controls [116]. Expansion of M1 macrophages were correlated with elevated anti-double-stranded DNA antibody production in murine model [59]. Increased mTORC1 activity makes T cells pro-inflammatory, however, it shift polarization pf macrophages towards M2 phenotype [59]. Surprisingly, M2 macrophages were found abundant in renal biopsy of lupus nephritis patients that raise the possibility of their contribution to inflammation [59].

## EFFECTS OF BIOLOGICS, SMALL MOLECULE INHIBITORS AND NSAIDS ON METABOLIC PATHWAYS

IL-1, IL-6 and TNF-α are all known to increase insulin resistance [117,118]. Studies have demonstrated that the blockade of those cytokines with biologic agents resulted in increased insulin sensitivity and better diabetic control [117,118]. IL-6 blockade therapy and JAK inhibitors are associated with increased LDL, HDL and triglycerides [117,119]. NSAIDs can reduce oxidative stress inside cells by reducing the production of ROS and IL-1 production and inhibit chemotaxis. In addition to inhibition of cyclooxygenase they also can activate PPAR (peroxisome proliferator activator receptor) α, increase expression of heat shock proteins and regulate the cytoskeleton through the Rho kinase pathway. [120]. In SLE T cells, genetically enforced increased expression of HRES-1/Rab4 causes the accumulation of oxidative stress-generating mitochondria [37] and activation of mTORC1 [121]. In turn, this makes this pathway an attractive target for small molecule inhibitors in SLE.

## CONCLUDING REMARKS

This brief review highlights several metabolic signatures, which have been identified as abnormal in autoimmune diseases and animal models of SLE, RA, psoriasis, and scleroderma. We summarized the existing metabolic targets used in DMARDS and highlighted several future potential targets to treat autoimmune diseases including mTOR, GCN2, IDO1, ARG 1, kynurenine, cysteine, S1P receptor and fatty acid enzymes. We also highlighted the therapeutic options of sirolimus, NAC, metformin, and halofuginone hydro bromide as well as some of the clinically observed benefits of fish oil in SLE patients. Ultimately research in metabolomics in SLE, RA, psoriasis, and scleroderma specifically has yielded treatment targets as well as safe therapeutic options for those patients. Targeting glycolysis via mTOR with sirolimus has clearly been shown in both humans and mice to ameliorate both surrogate markers (such as anti dsDNA) as well as composite validated disease measures such as SLEDAI and BILAG. While balancing energy production between OXPHOS and glycolysis has been studied extensively, maintenance of a reducing environment by the PPP and other antioxidant pathways if far less understood. Continued work looking at cell type-specific activation of mTOR complexes and introduction of mTORC2 or dual mTORC1/mTORC2 complex inhibitors is likely to yield more therapeutic options for SLE patients.

## Figures and Tables

**Table 1.** Metabolic pathways targeted by conventional and experimental drugs in autoimmune diseases.

Pathway	Molecular Target	Drug	Disease	Ref
Glycolysis	GCR	Corticosteroid	SLE, RA, PsA	[13,14]
	PGI	2 DG	SLE	[15]
Purine metabolism	DHFR, ATIC	Methotrexate	RA, PsA	[16]
	TPMT	Azathioprine	RA, SLE	[17,18]
	IMPDH	Mycophenolate	SLE	[19]
Pyrimidine metabolism	DHODH	Leflunomide	RA	[20]
GSH	Cysteine	NAC	SLE	[21]
			RA, CIA	[22,23]
			Sjogrens	[24]
Mitochondria	Complex I	Metformin	SLE, CIA	[15,25]
	Complex II	MitoQ	SLE, EAE	[26,27]
	mitoNEET	Pioglitazone	SLE	[26,28]
Autophagy	mTORC1	Rapamycin	SLE	[29,30]
			Lupus nephritis	[31,32]
			SSc	[33]
			Sjogrens, RA	[34,35]
	Lysosome	HCQ	RA, SLE	[36]
Mitophagy	Drp1	3-PEHPC	SLE	[37]
Sphingolipid signaling	S1P receptor	Fingolimod	SLE, MS, IBD	[38]
		DHS1P	SSc	[39]

Abbreviations: IBD = Inflammatory bowel disease, SSc = Systemic sclerosis, PsA = Psoriatic arthritis, CIA = collagen induced arthritis, EAE = experimental auto immune encephalitis, HCQ = Hydroxychloroquine, PGI = phosphor glucose isomerase, GCR = Glucocorticoid receptor, TPMT = Thio purine methyl transferase, IMPDH = inosine monophosphate dehydrogenase, DHODH = Dihydroorotate dehydrogenase, DHFR = Dihydrofolate reductase, ATIC = AICAR transformylase/IMP cyclohydrolase, MitoQ = Mitoquinone, 3-PEHPC = 2-(3-pyridinyl)-1-hydroxyethylidene-1,1-phosphonocarboxylic acid.

## ABBREVIATIONS

SLE: systemic lupus erythematosus
RA: rheumatoid arthritis
PsA: psoriatic arthritis
DMARDs: Disease modifying anti rheumatic drugs
mTOR: mammalian target of rapamycin
AMPK: adenosine monophosphate activated protein kinase
AhR: aryl hydrocarbon receptor
GCN2: general control non derepressible 2
NADH: reduced nicotinamide dinucleotide
FADH: reduced flavine adenine dinucleotide
NADPH: reduced nicotinamide adenine dinucleotide
ATP: adenosine triphosphate
PPP: pentose phosphate pathway
3-PEHPC: 2-(3-pyridinyl)-1-hydroxyethylidene-1,1-phosphonocarboxylic acid
CD4: cluster differentiation 4
FLS: fibroblast like synoviocytes
2DG: 2 deoxyglucose
HK2: hexokinase 2
GLUT1: glucose transporter 1
LDH: lactate dehydrogenase
HIF-1alpha: hypoxia inducible factor 1 alpha
OXPHOS: oxidative phosphorylation
TCA: tricarboxylic acid
PPAR: peroxisome proliferator activated receptor
IL: interleukin
MAVS: mitochondrial antiviral signaling protein
Drp1: dynamin related protein 1
LN: lupus nephritis
SLEDAI: SLE disease activity index
BILAG: british isles lupus activity group
pDC: plasmacytoid dendritic cells
ANA: anti-nuclear antibody
TAL: transaldolase
GSH: reduced glutathione
NAC: *N*-acetylcysteine
ROS: reactive oxygen species
NOX2: NADPH oxidase 2
IDO: indoleamine-2,3-dioxygenase
ArG1: arginine 1
HMG CoA: 3-hydroxy-3-methyl-glutaryl-coenzyme A
S1P: sphingosine 1 phosphate
CTLA 4: cytotoxic T lymphocyte associated protein
TGF Beta: transforming growth factor beta
KYN: kynurenine
PFK: phosphofructokinase
STING: stimulator of interferon genes
IFN: interferon
ISG: interferon signature
cGAS-cGAMP synthase: cGAMP cyclic guanosine monophosphate–AMP
PFKFB3: 6-phosphofructo-2-kinase/fructose-2,6-bisphosphatase isoenzyme 3
AIM2: absent in melanoma 2
IL-18: interleukin-18
IL-1b: interleukin-1b
MRE11A: meiotic recombination 11
NLRP3: NLR family pyrin domain containing 3
